# Malaria hotspots explained from the perspective of ecological theory underlying insect foraging

**DOI:** 10.1038/s41598-020-78021-x

**Published:** 2020-12-08

**Authors:** Yared Debebe, Sharon Rose Hill, Habte Tekie, Sisay Dugassa, Richard J. Hopkins, Rickard Ignell

**Affiliations:** 1 Department of Zoological Sciences, Addis Ababa University, PO. Box 1176, Addis Ababa, Ethiopia; 2 Unit of Chemical Ecology, Department of Plant Protection Biology, Swedish University of Agricultural Sciences, Alnarp, Sweden; 3 Aklilu Lemma Institute of Pathobiology, Addis Ababa University, PO. Box 1176, Addis Ababa, Ethiopia; 4 Natural Resources Institute, University of Greenwich, London, UK

**Keywords:** Ecology, Epidemiology

## Abstract

Hotspots constitute the major reservoir for residual malaria transmission, with higher malaria incidence than neighbouring areas, and therefore, have the potential to form the cornerstone for successful intervention strategies. Detection of malaria hotspots is hampered by their heterogenous spatial distribution, and the laborious nature and low sensitivity of the current methods used to assess transmission intensity. We adopt ecological theory underlying foraging in herbivorous insects to vector mosquito host seeking and modelling of fine-scale landscape features at the village level. The overall effect of environmental variables on the density of indoor mosquitoes, sporozoite infected mosquitoes, and malaria incidence, was determined using generalized linear models. Spatial analyses were used to identify hotspots for malaria incidence, as well as malaria vector density and associated sporozoite prevalence. We identify household occupancy and location as the main predictors of vector density, entomological inoculation rate and malaria incidence. We propose that the use of conventional vector control and malaria interventions, integrated with their intensified application targeting predicted hotspots, can be used to reduce malaria incidence in endemic and residual malaria settings.

## Introduction

Malaria prevention and control strategies have resulted in a remarkable reduction of malaria mortality and morbidity throughout most of sub-Saharan Africa over the past two decades^1^. The implementation of e.g., indoor residual spraying, long-lasting insecticide treated bed nets and rapid detection tests for malaria parasites, however, are not sufficient to eliminate malaria at a local level^2^. Climatic factors and transmission seasons are considered important drivers of malaria epidemiology for implementing integrated vector management (IVM) tools over wider geographic areas, *e.g*., wards, counties, and countries^3^. To develop a more robust and targeted intervention approach, which considers heterogeneity at the local level, however, there is a need to consider the eco-epidemiological settings of malaria transmission over much finer geographical scales^4–6^.

Fine scale spatial heterogeneity of malaria incidence and vector density has been demonstrated in areas with different transmission magnitudes in many malaria-endemic countries^7–9^. Pockets of high malaria incidence, hotspots, have been described at the village level^6,10,11^. Hotspots constitute the major reservoir for persistent malaria transmission and are associated with higher vector density, sporozoite prevalence and malaria incidence than neighbouring areas^9,12^. Malaria transmission in these hotspots has been suggested to be primarily driven by local environmental conditions, e.g., proximity to vector breeding sites^10,13,14^, vegetation cover^15,16^, housing structure and condition^17–19^, bed net use^20,21^, and household occupancy^22^. To date, however, there is no consensus as to which factors underlie the formation of hotspots at the village level, and the identification of these is crucial to attain the Sustainable Development Goal of zero malaria incidence level in a given area^23,24^.

Female mosquitoes, like many insects, seek a range of different resources over the course of their lifespan, and the trade-off between the benefits of e.g., sugar meals and blood meals are critical to the overall fitness of a female seeking to gain such resources^25^. Moreover, these trade-offs can be modulated with time and physiological state, e.g., as the female ages, the likelihood of taking a blood meal over that of a sugar meal increases^25,26^. Resources are not evenly distributed within space and time, and the theoretical framework describing the observed spatial heterogeneity of blood feeding resources in the mosquito landscape is currently lacking. However, there is a plethora of theories based on insect-plant interactions that may be adapted for this purpose^27^. Jones^28^ emphasized that search outcomes in herbivorous insects are dependent on behavioural patterns within species, which can be broadly categorized into the resource concentration hypothesis^29^ or the edge effect^30^. The resource concentration hypothesis postulates that searching individuals are likely to locate and remain in stands with a high host density, and the edge effect favours resources being more extensively used in sparse patches, and importantly more on the edge than in the centre of those patches. The edge is defined as the abrupt transition between habitats differing in quality, affecting the performance of individuals^31^. A patch can be defined as a relatively homogenous nonlinear area that differs from its surroundings^32^ and for mosquitoes, we may regard the assembly of blood hosts in a community, either at the household or village level, as a patch, and the homes placed on the outskirts of a community as on the edge of that patch.

In this study, we examine the distribution of indoor resting and host-seeking mosquitoes, and the resulting spatial occurrence of malaria, within village communities to assess whether residents associated with individual households are more likely to be exposed to blood feeding by mosquitoes within village communities. Spatial clustering and distribution of mosquito activity and malaria incidence, as well as the distribution of sporozoite-infected vectors, were mapped, and hotspots/coldspots of malaria incidence and vector densities identified in two rural villages in southern Ethiopia. To identify the direct and indirect factors, e.g., household location and occupant density, underlying these hotspots/coldspots, we created and tested local landscape models. Local environmental conditions were categorized and their influence on mosquito density and malaria incidence was determined. We conclude that an increased understanding of vector ecology and biology is required to identify novel strategies that can complement current IVM.

## Results

### Local spatial clustering of malaria mosquitoes

**Figure 1:**
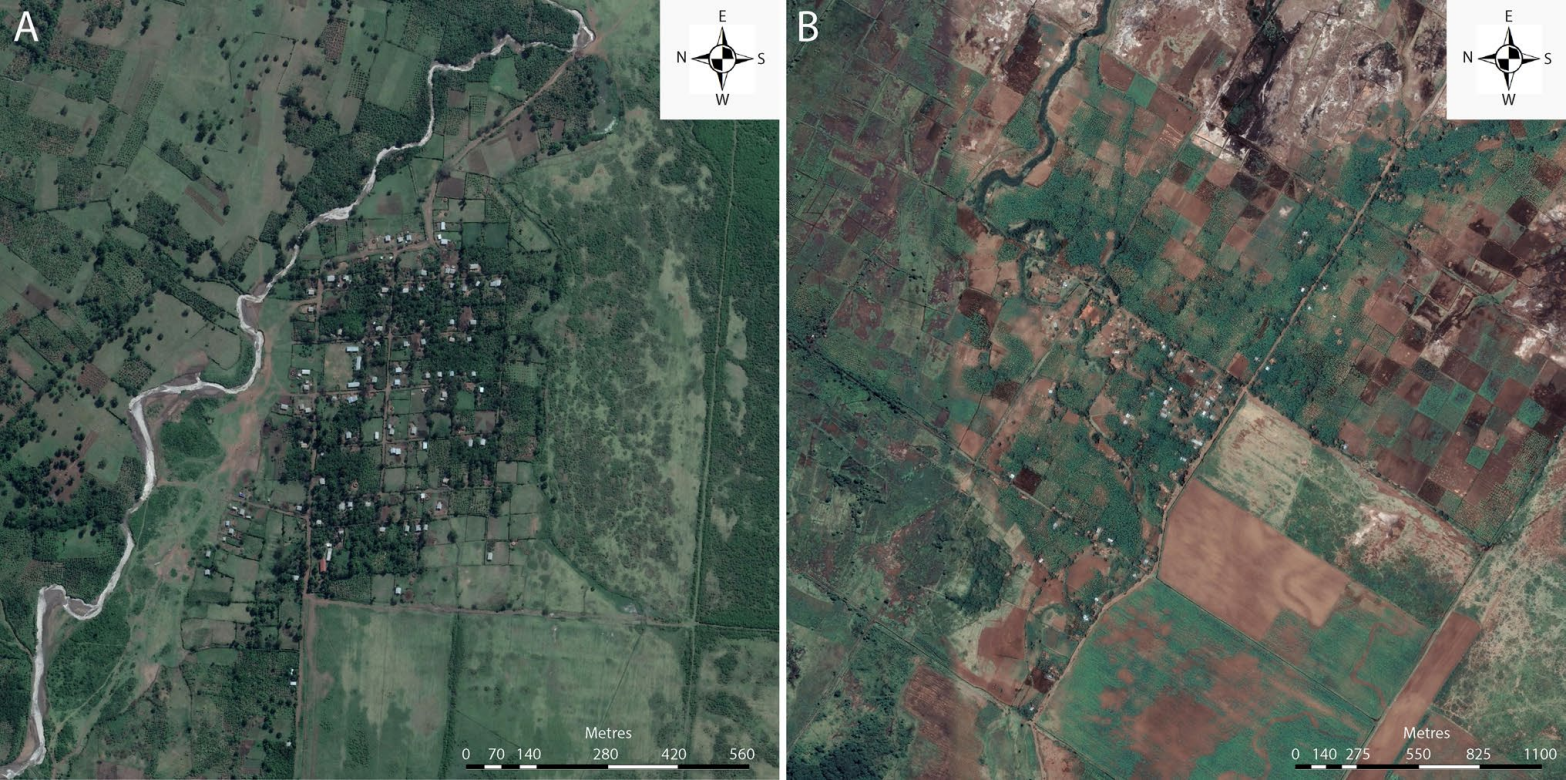
Aerial images of the two study villages, Abulo (**A**) and Magge (**B**). The two rural villages are situated within similar agricultural landscapes. Aerial images are taken at different times of the year, which is seen in the difference in crop cover in the two villages. The scale bars indicate distance (m). The aerial images were obtained using the open software Google Earth Pro (Version 7.3.3.7786).

**Figure 2:**
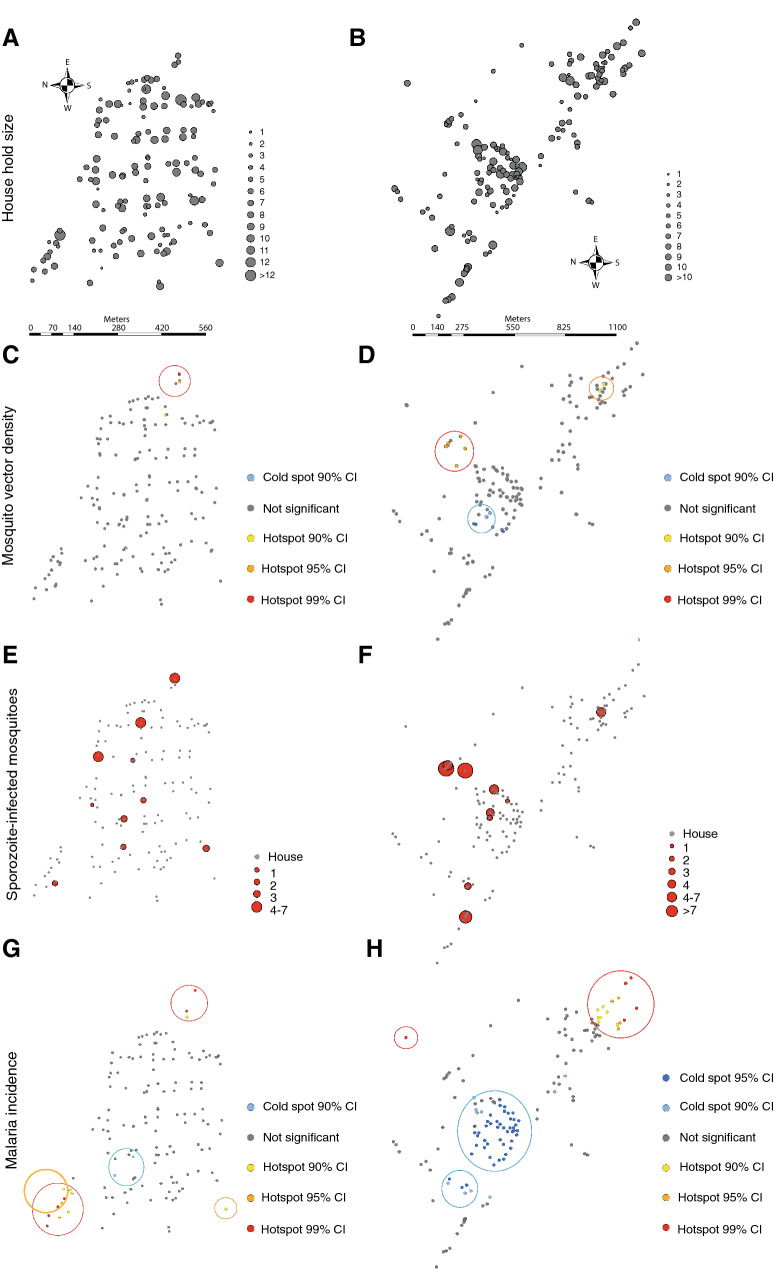
Living on the edge increases malaria incidence. The distribution of households and occupant density in Abulo (**A**) and Magge (**B**), in which the size of the circles indicate the number of inhabitants in each household. The scale bars indicate distance (m). Clustering of malaria mosquitoes generated from hotspot analyses in Abulo (**C**) and Magge (**D**) is shown. Cold- and hotspots are indicated with 90%, 95% and 99% confidence intervals (CI). The distribution and abundance of sporozoite-infected mosquitoes is mapped for Abulo (**E**) and Magge (**F**). The size of the circles indicates the number of sporozoite-infected mosquitoes. The clustering of malaria infected people and people with lower risk of getting the infection is revealed by the hotspot analyses for Abulo (**G**) and Magge (**H**). The coloured rings indicate the different significance levels of hot- and coldspots, with 90%, 95% and 99% CI.

From a total of 750 sampling nights in each village, 2517 and 2377 mosquitoes were caught/collected indoors and outdoors in Abulo and Magge (Fig. 1), respectively (Table 1). Clustering analyses revealed that the three most abundant *Anopheles* species aggregated in and around a few houses located close to one another near the edge of each village (Fig. 2A–D). In Abulo, one hotspot was identified at the eastern edge (Gi^* ^Z ≥ 1.96, Gi^* ^P ≤ 0.05), whereas the clustering of mosquitoes in other parts of the village was not statistically significant (Gi^* ^Z < 1.96, Gi^* ^P > 0.05; Fig. 2C). Similarly, in Magge, one statistically significant (Gi^* ^Z ≥ 1.96, Gi^*^ P ≤ 0.05), and one nearly significant hotspot (Gi^* ^Z < 1.96, Gi^* ^P < 0.1) were identified at the northern and eastern edges of the village, respectively, in which the majority (52.6%) of the mosquitoes were collected (Fig. 2D). In contrast, a coldspot was detected in the centre of Magge (Gi^* ^Z > 1.96, Gi^* ^P < 0.05; Fig. 2D).

**Table 1:** The species of *Anopheles* and the total number of mosquitoes caught in indoor and outdoor Centre of Disease Control light traps and collected following pyrethrum spray treatment.

*Anopheles* species	Abulo			Magge		
	CDC light traps		PSC (N)	CDC light traps		PSC (N)
	Indoor (N)	Outdoor (N)		Indoor (N)	Outdoor (N)	
*An. arabiensis*	787	108	761	1286	145	388
*An. pharoensis*	381	135	3	226	73	0
*An. ziemanni*	120	86	0	102	115	1
*An. demeilloni*	19	28	19	21	3	13
*An. pretoriensis*	20	39	0	3	1	0
*An. tenebrosus*	2	3	0	0	0	0
*An. garnhami*	1	0	0	0	0	0
*An. squamous*	1	0	0	0	0	0
*An. cinereus*	0	1	0	0	0	0
*An. natalensis*	0	3	0	0	0	0
Total	1331	403	783	1638	337	402

### Distribution of sporozoite-infected vectors

**Figure 3:**
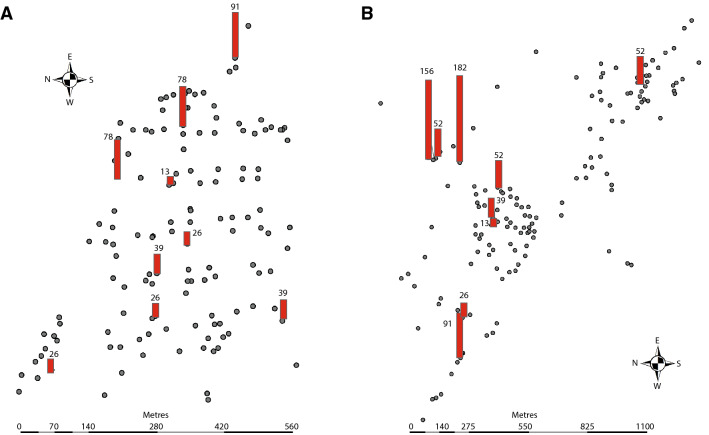
Map showing the number of infectious bites an individual receives per year (the red bars) and the households in the study areas (the grey dots). The scale bars indicate distance (m).

While spatial clustering analysis is not possible for this variable, there being only ten locations per village rather than the 30 required, the geographical location of the 83 sporozoite-infected mosquitoes was mapped for Abulo (Fig. 2E) and Magge (Fig. 2F). While the majority of the sporozoite-infected mosquitoes were caught in houses at the edge of the villages (Fig. 2E,F), the proportion of sporozoite-infected mosquitoes at each sampling point was dependent on the density of the vectors, as indicated by the different entomological inoculation rates (EIR) in houses located at the edge and the centre of the villages (Fig. 3). In Abulo, the chance of receiving an infectious bite doubled in houses located at the edge compared to those at the centre: occupants of houses at the village edge experienced the risk of 50.23 infectious bites per person per year (ib/p/y) as opposed to those at the centre who risk 25.78 ib/p/y. Similarly, in Magge, the likelihood of an occupant receiving an infectious bite at the village edge was three times higher (82.44 ib/p/y) at the edge compared to the centre (26.21 ib/p/y).

### Local spatial clustering of malaria incidence

Malaria incidence indicated the presence of high and low malaria risk areas, hotspots and coldspots, respectively, in both villages. In Abulo, two hotspots were identified (Gi^* ^Z > 1.96, Gi^* ^P < 0.05), which accounted for 35.3% of the malaria incidence, and were located at opposite ends of the village on the edge, along with a close to significant hotspot at the southwestern edge of the village (Gi^* ^Z < 1.96, Gi^*^ P < 0.1; Fig. 2G). A close to significant coldspot (Gi^* ^Z < 1.96, Gi^* ^P < 0.1) around the centre of the village was detected in Abulo (Fig. 2G). Similar to Abulo, a higher incidence of malaria was found clustered at the remote edge of the village in Magge (Gi^*^ Z > 1.96, Gi^*^ P < 0.05), with a coldspot clustered at the centre of the village (Gi^* ^Z < 1.96, Gi^*^ P < 0.05; Fig. 2H).

### Environmental factors differentially affect the density of indoor *Anopheles*

A total of 4154 *Anopheles* mosquitoes, belonging to eight species, were captured and collected indoors using CDC light traps and PSC, respectively (Table 1). Approximately 98% of the mosquitoes were identified as *An. arabiensis*, *An. pharoensis* and *An. ziemanni* (Table 1). For the analysis, data for these three species were pooled, as the number of *An. pharoensis* and *An. ziemanni* were relatively low, and the other species removed. The overall regression analysis demonstrated that none of the environmental variables significantly affected the indoor density of these species (Supplementary Table S1), likely due to the inherent high variation when comparing between villages. As a result, subsequent analyses were conducted at the village level, which revealed that the environmental variables differentially affected the indoor mosquito density. In Abulo, the overall GLM analysis revealed that the density of mosquitoes indoors was significantly affected by the household size, bed net use and the interaction of bed net use and house location in the village (Supplementary Table S2) with an AIC value of 258. Backward elimination of non-significant variables resulted in the final simplest model, with a reduced AIC value of 227, indicating that household size, bed net use and the interaction of bed net use and house location in the village significantly affected the indoor density of *Anopheles* mosquitoes (Table 2).

**Table 2:** Statistical summary of the effect of environmental variables on the density of indoor mosquitoes at the village level following stepwise backward selection and removal of non-significant independent variables.

Term	Estimate	Std error	Wald χ^2^	Prob > χ^yyy^
**Abulo**				
Intercept	2.70	0.65	17.21	< 0.0001
House location in the village (center/edge)	0.45	0.48	0.89	0.34
Household size (No of occupants)	0.19	0.079	5.55	0.019
Net use (proper use/no use)	1.20	0.39	9.60	0.0019
Net use * House location in the village	− 1.81	0.64	7.93	0.0049
**Magge**				
Intercept	4.31	0.91	22.54	< 0.0001
Wall condition (poor/good)	1.60	0.49	10.68	0.0011
House location in the village (center/edge)	0.70	0.72	0.93	0.34
Net use (proper use/no use)	− 0.59	0.71	0.71	0.40
Net use * House location in the village	2.30	0.98	5.27	0.022
Roof condition (poor/good)	− 1.47	0.73	4.04	0.045

In Magge, the overall GLM analysis demonstrated that the housing conditions, including door, wall and roof condition, household size, bed net use, and the interaction between bed net use and location of the house in the village significantly affected the indoor density of mosquitoes (Supplementary Table S3; AIC = 282). The final model (AIC = 234) revealed that there was significant effects of wall and roof condition, as well as significant interaction between bed net use and location of the house in the village. This interaction demonstrates that the density of indoor *Anopheles* mosquitoes is higher in houses that did not use bed nets properly and are located at the edge of the village.

### Environmental factors do not affect the density of sporozoite-infected vectors

Out of the 2898 mosquitoes caught in the indoor CDC traps in both villages, 83 were found to be sporozoite positive. The number of sporozoite-infected mosquitoes was not significantly affected by any of the environmental factors included in the regression models, both at the overall and village levels.

### Environmental factors affect malaria prevalence

Blood samples were taken from the general population with a diverse age distribution. The majority of blood samples were taken from children between the ages of 5 and 15 years (55%). Teenagers and adults of ages 16 and above (25%), children aged 1–4 years (19%), and infants less than one year old made up the rest. Out of the 1894 persons sampled, a total of 64 individuals were found to be infected with *Plasmodium* parasites during the repeated cross-sectional surveys in both villages. The overall prevalence of malaria in Abulo was 3.53% (34/964) and 3.23% (30/930) in Magge. The overall GLMM model revealed that the number of malaria cases in the two villages combined was significantly affected by the interaction of house location and household size (GLMM; F = 5.50; P = 0.021), with people living in houses located at the edge of the villages, with a higher number of occupants (Fig. 2A,B), being more likely to become infected with malaria. Separate analyses provided further support for these findings in both villages. In Abulo, when all of the housing and environmental conditions were included in the GLMM model, household size was found to significantly affect the number of *Plasmodium*-infected individuals (AIC = 267; Supplementary Table S4). Stepwise backward elimination of independent variables with the highest P-value resulted in the final model, in which house location in the village and household size both affected malaria prevalence resulting in AIC value of 218 (Table 3). In Magge, only the location of the house in the village in both the overall (AIC = 259; Supplementary Table S5) and the final model (AIC = 230; Table 3) was found to be a significant predictor of malaria prevalence.

**Table 3:** Statistical summary of the effect of environmental variables on the incidence of malaria at the village level, following stepwise backward selection and removal of non-significant independent variables.

Variable	Estimate	Std error	t ratio	Prob >|t|
**Abulo**				
Intercept	− 0.018	0.099	− 0.19	0.85
Household size (no of occupants)	0.047	0.015	3.04	0.0029
Breeding site within 50 m radius (present/absent)	− 0.086	0.062	− 1.38	0.17
House location in the village (center/edge)	− 0.082	0.041	− 2.02	0.045
**Magge**				
Intercept	0.20	0.043	4.54	< 0.0001
House location in the village (center/edge)	− 0.14	0.043	− 3.18	0.0018

## Discussion

Insect-plant interactions provide the theoretical framework by which vector density and malaria prevalence can be modelled in a heterogeneous local landscape. By testing local landscape models within two rural villages in southern Ethiopia, we identify household size and location within the village as the main predictors of vector density and malaria prevalence, supporting the resource concentration hypothesis and edge effect, respectively^29,30^. By extending the analysis to sporozoites, we further identify the edge of the villages as at increased risk for obtaining malaria through enhanced EIR. The efficacy and ability to implement malaria interventions based on the identification of hotspots is controversial, and has been said to rely heavily on the ease of hotspot identification, persistence and the level of landscape heterogeneity^23,33,34^. We argue that hotspot analysis coupled with landscape models based on direct and indirect effects on mosquito density, and in the light of established ecological frameworks, can inform malaria intervention strategies at the local level using generalizable criteria. Thus, we may avoid time-consuming and low sensitivity methods of identifying people with increased exposure to infectious mosquitoes.

Through the regression models, hotspot analyses and distribution of EIRs in two rural villages over fifteen consecutive months, we identify the edge of the villages as areas of increased risk for malaria as a consequence of high, overlapping populations of both mosquitoes and people. In contrast, people at the centre of the villages experience a reduced risk of exposure. This effect is likely not due to heterogeneity in the quality within the patch, in terms of blood meals, but rather to the heterogeneity associated with the risk connected with foraging in the centre or at the edge of the patch^31^. It is important to note that in the villages under discussion, the livestock come into the village every night, and disperse throughout the village to household associated cattle pens/sheds. Thus, alternate host availability is likely not a contributing factor to the observed edge effect. While the limited number of studies investigating malaria hotspots at the within-village level did not consider the mosquito density and household location, these still demonstrate a clear impact of the edge effect on the spatial clustering of malaria incidence^6,10,14^. Although entomological detection of hotspots may currently be logistically unattractive and hampered by poorly standardized sampling strategies, the implementation of standardized entomological sampling strategies, including CDC light traps and PSC, which are based on the knowledge of the edge effect and the resource concentration hypothesis on vector activity, can provide a robust indicator of malaria hotspots.

The host-seeking behaviour of female mosquitoes is influenced by various factors, including the dispersal ability of the mosquitoes, as well as the availability, density and distribution of hosts^35–38^. Host-seeking mosquitoes are known to leave their resting sites, situated either in tall vegetation at some distance from the households, or in the agricultural landscape surrounding the village, to gain access to their blood hosts^15,39–41^. Household occupancy is known to influence indoor vector densities, and thereby affect mosquito dispersal, and by extension, the distribution of human biting risk and malaria transmission within communities^22,42,43^. Moreover, the overall directional movement of mosquitoes within villages is influenced by the spatial distribution and demographic composition of households in these villages^44^. As a result, households with high occupancy may form pockets (patches) of high transmission of mosquito-borne diseases^45^, as demonstrated in this study. However, the fine-scale and within-village clustering of vector densities in this study does not appear to overlap with the clusters of houses with high occupancy, except at the village edge. In fact, houses with high occupancy within the centre of both study villages are coldspots for malaria incidence and vector presence, indicating that the indirect effect of the location can mitigate the direct effect of household occupancy. Thus, census data alone does not accurately reflect malaria vector hotspots, as previously suggested^22^. Regardless of the difference in topography between Abulo and Magge, this study demonstrates that the same fine scale landscape features modulate the spatial relationships underlying malaria transmission.

In this study, we identify the landscape features that are involved in forming vector and malaria hotspots within villages. We propose that maintaining or scaling up conventional vector control and malaria interventions, in combination with the targeted intensification of these, or other novel controls, in predicted hotspots, can be implemented to reduce malaria incidence. Future studies concerning control measures that investigate the effects of intensified interventions at the edge of the village, particularly in the context of regional intervention programmes, would clarify, and likely validate, the generalisability of the results presented here. One complicating factor in studying the edge effect is in monitoring the pre-alighting behaviour of the vector, which in the case of mosquitoes requires ingenuity and innovation^28^. In order to increase the resolution of the effect of the edge on vector density, a similar study could be conducted by monitoring along transects through the village, to assess the vector density over time throughout the intervention. This can be conducted either through classical trapping, or by analysing the real-time dispersal of the mosquitoes by using high resolution entomological lidar^46^. Previously, the implementation of hotspot-targeted vector control strategies was seen as highly labour intensive with variable outcomes^33^. With the implementation of the general rules identified in this study, there would a minimal increase in labour and cost associated with the treatment of predicted hotspots, in addition to the current maintenance control efforts.

## Methods

### Description of the study area

The study was conducted in two rural villages located near to Arba Minch, ca. 500 km south of Addis Ababa. Abulo (6° 03′ 48″ N, 37° 35′ 30″ E) is located 6 km northeast of Arba Minch and is an isolated village, more than 2 km away from the nearest settlement and main road, with 134 clustered households and a total population of ca. 900. The village is situated 1.5 km from Lake Abaya, and is well drained by irrigation canals, with an adjacent river. Magge (5° 51′ 49″ N, 37° 29′ 32″ E) is located 22 km south of Arba Minch. The total population of this isolated (> 2 km away from nearest settlement and main road) and clustered village is ca. 700 residents. The village is situated 1.7 km from Lake Chamo. The houses in both villages are roofed with either grass thatch or corrugated iron sheets and have mud walls. Residents of both villages have similar socio-economic status with noticeable poverty. Banana plantations, livestock rearing, fishing and subsistent farming are the common economic activities sustaining the livelihood of the residents. In addition to the economic benefit, keeping cattle and other domestic animals is a traditional heritage exercised by the majority of the residents. Farmlands are usually located at the outskirts of the villages, where maize is predominantly harvested. Malaria is a common problem in both villages. The presence of perennial water and the proximity of both villages to lakes make both villages suitable for proliferation of mosquito vectors throughout most of the year (Fig. 1A,B).

### Characterization of environmental factors

In order to identify potential environmental risk factors underlying the clustering of malaria vectors, with and without sporozoite infection, as well as of malaria-infected people, at household and village levels, and a survey of each house and the surrounding environment was conducted and characterized. The location of each house was georeferenced using a global positioning system (GPS; Garmin eTrex 10, Garmin International Inc, USA). Data on the location of the house in the village; housing condition (type, construction material, presence or absence of eaves, door and window condition, roofing and wall materials and condition); number of residents; cooking habits (inside or outside houses); bed net use; presence or absence of alternate hosts outdoors; and presence of mosquito breeding sites within 50 m in the two study villages were recorded.

### Entomological monitoring

Monthly sampling of malaria mosquitoes was conducted in the two study villages from January 2018 to March 2019. A systematic random sampling approach was used to select 30 houses in each village for the subsequent sampling of malaria mosquitoes. Ten of the houses were used for indoor collections with Centers for Disease Control and Prevention (CDC) light traps (BioQuip Products, Inc, CA, USA), while ten other houses were selected for pyrethroid spray collections (PSC; Mobile insecticide, Fujian Quanzhou Gaoke Daily Chemical Manufacturing Co Ltd, China). In addition, ten houses were used for outdoor collections using CDC light traps. To optimize spatially balanced sampling, the two study villages were each divided into three blocks, comprising two at the edges and one at the centre. Six houses from the two edge blocks and four houses from the central block were randomly selected in each study village. The selected houses were similar in structure and construction materials. In the case in which a house was different in structure, the neighbouring house with similar features was chosen.

The indoor CDC light traps were hung next to the feet of an occupant, sleeping under an insecticide-treated bed net, approximately one meter above the ground, following the protocol outlined by the WHO^47^, while outdoor CDC traps were hung next to the houses approximately 50 cm above the ground. The traps were operated from 6:00 PM to 6:00 AM and the indoor traps collected over three alternate nights, resulting in 30 CDC trap nights per village per month, while the outdoor traps were operated once a month. The PSC collections were conducted once a month according to the WHO specifications^47^. Before spraying, occupants were requested to leave the house. Utensils used for food, drinking water and clothes were taken out of the houses, and openings in the walls and doors were covered with cloth in order to prevent the mosquitoes from escaping during spraying. The floor of the house was covered with white sheets. Knocked down female *Anopheles* mosquitoes were then collected and preserved individually in microfuge tubes (1.5 ml) containing silica gel desiccant.

The collected mosquitoes from all sampling methods were sorted according to their physiological status, as unfed, blood-fed, semi-gravid or gravid, and morphologically identified into species using standard keys^48,49^. All female anopheline mosquitoes were individually preserved in labelled tubes containing silica gel and stored at room temperature, until further processing. Mosquitoes in the *An. gambiae* species complex were considered as *An. arabiensis*, since no other species in the complex has been recently recorded in the area^15,50^.

### Detection of Plasmodium sporozoite-infected mosquitoes

All *An. arabiensis*, *An. pharoensis,* and *An. ziemanni* caught in indoor CDC light traps were analysed for the presence of *Plasmodium falciparum* and *P. vivax* circumsporozoite proteins (CSP). This was done using enzyme linked immunosorbent assay (ELISA) following the procedure outlined by Wirtz et al*.*^51^.

### Cross-sectional parasitological studies

Four phase cross-sectional studies were conducted in each village, in January, August, November 2018 and March 2019. The participating households were randomly selected and then visited. The geographic location of each house visited was recorded using a GPS. A total of at least 215 people for Abulo (N = 897) and 201 people for Magge (N = 701) were included for the cross-sectional parasitological examination during each period. The sample size was determined based on a prior study on the prevalence of malaria in the district^52^, which reported that the overall malaria prevalence was 24.3%. To determine the sample size, the finite population formula was used: n' = [NZ^2^ P (1-P)] **/** d^2^ (N-1) + Z^2^P (1-P), where n' is the sample size; N is the population size; Z is the Z statistic for a level of confidence; P is the expected proportion of people carrying *Plasmodium* parasites; and d is the level of confidence^53^.

Thick and thin blood smears were prepared on clean microscopic slides, by puncturing the ‘ring finger’ of the study participants with a sterile lancet, following WHO standard guidelines^54^. Rapid diagnostic test (RDT) kits were also used for the immediate detection of positive cases. Informed consent was obtained from study participants and parents of children involved in the study prior to taking blood samples. All malaria positive cases were treated according to the Ethiopian Ministry of Health Guidelines^55^. A research permit was obtained from the institutional review board of the College of Natural and Computational Sciences, Addis Ababa University (CNSDO/284/08/2016) and from the Arba Minch Zuria District Health Office (AZWHO/1163/2). All methods were performed in accordance with the relevant guidelines and regulations.

### Spatial analysis

To assess the local spatial autocorrelation of malaria vectors and *Plasmodium*-infected individuals, the Getis-Ord Gi statistics was used. The Getis-Ord Gi statistics is effective in identifying pockets of high and low incidence points referred to as hotspots and coldspots, respectively^56^. Hotspots and coldspots for the density of mosquito vectors and malaria cases were identified, and statistical significance was determined at Gi^*^ P values of 0.05 or less. Sporozoite-infected mosquitoes and the annual EIR were mapped based on their respective value. Graduated symbols and bars indicate the variation in the number of sporozoite-positive mosquitoes and the annual EIR, respectively, among the houses. ArcGIS (v. 10.3, ESRI, USA) was used to produce all the maps.

### Environmental risk factor analysis

The effect of housing structure and other environmental factors on the density of indoor *Anopheles*, as well as the prevalence of sporozoites, was modelled using negative binomial regression of the generalized linear model (GLM; JMP Pro version 13 SAS Institute Inc., Cary, NC, USA). In contrast, repeated measures generalized linear mixed model (GLMM) was used to model the effect of housing structure and local environmental factors on the number of *Plasmodium*-positive individuals. To determine the overall effect of the environmental variables on the overall density of indoor mosquitoes, sporozoite-infected mosquitoes and malaria prevalence, the regression analysis was first conducted by pooling the data from both study villages. This model determined that the village was a non-significant factor, and therefore subsequent analyses were conducted at the village level. The regression analysis was first conducted using all the predictor environmental factors for each village to generate the overall models. Selection of the final model was based on the Akaike’s information criterion (AIC) in which the most parsimonious model with the lowest AIC value. Stepwise backward selection and removal of the independent variables with the highest p-values from the overall model were conducted until the AIC value of the model was minimized. All independent variables and interactions between independent variables retained in the final models are presented in Tables 2 and 3. The entomological inoculation rate (EIR) was estimated from the CDC indoor captures using the formula: 1.605 × (number of circumsporozoite-positive ELISA results from CDC light trap / number of mosquitoes tested) × (number of mosquitoes collected from CDC light trap / number of catches) × 365 days^57^.

## Supplementary information

Supplementary Information

## Data Availability

All data generated or analysed during this study are included in this published article.
