# Supplementary material for: Malaria hotspots explained from the perspective of ecological theory underlying insect foraging
Source: Sci Rep. 2020 Dec 8;10:21449. doi: 10.1038/s41598-020-78021-x (PMC7722757; doi:10.1038/s41598-020-78021-x)
Supplement: Supplementary file 1 — Supplementary Information [file 41598_2020_78021_MOESM1_ESM.docx]

**Supplementary Table 1:** Statistical summary of the effect of housing conditions and local environmental variables on the density of indoor mosquitoes in Abulo and Magge.

| **Variable** | **Wald Chi-Square** | **Prob > Chi-Square** |
| --- | --- | --- |
| House location in the village (center/edge) | 2.20 | 0.14 |
| Net use (proper use/no use) | 0.53 | 0.46 |
| Wall condition (good/poor) | 0.41 | 0.52 |
| Livestock ownership (yes/no) | 0.37 | 0.54 |
| Household size (No of occupants) | 0.12 | 0.73 |
| Cooking inside houses (yes/no) | 0.11 | 0.74 |
| Breeding site within 50 m radius (present/absent) | 0.073 | 0.79 |
| Door (curtain/wood/metal) | 1.020 | 0.79 |
| Village | 0.026 | 0.87 |
| Door condition (good/poor) | 0.0091 | 0.92 |
| Eaves (present/absent) | 0.00032 | 0.99 |
| Roof condition (good/poor) | 0.00022 | 0.99 |

**Supplementary Table 2**: Statistical summary of the effect of housing conditions and local environmental variables on the density of indoor *Anopheles* mosquitoes in Abulo.

| **Variable** | **Wald χ^2^** | **Prob > χ^2^** |
| --- | --- | --- |
| Net use (proper use/no use) | 12.02 | 0.0005 |
| Net use * House location in the village | 11.07 | 0.0009 |
| Household size (No of occupants) | 4.37 | 0.037 |
| Door material (curtain/wood/metal) | 3.36 | 0.067 |
| House location in the village (center/edge) | 2.51 | 0.11 |
| Wall condition (good/poor) | 1.32 | 0.25 |
| Livestock ownership (yes/no) | 1.07 | 0.30 |
| Household size * House location in the village | 1.01 | 0.31 |
| Door condition (good/poor) | 0.91 | 0.34 |

**Supplementary Table 3:** Statistical summary of the effect of housing conditions and local environmental variables on the density of indoor *Anopheles* mosquitoes in Magge.

| **Variable** | **Wald  χ^2^** | | **Prob > χ^2^** |
| --- | --- | --- | --- |
| Wall condition (good/poor) | 20.13 | <0.0001 | |
| Net use * House location in the village | 17.61 | <0.0001 | |
| Roof condition (good/poor) | 12.06 | 0.0005 | |
| Household size (No of occupants) | 7.41 | 0.0065 | |
| Net use (proper use/no use) | 6.69 | 0.0097 | |
| Door condition (good/poor) | 5.01 | 0.025 | |
| Breeding site within 50 m radius (present/absent) | 2.69 | 0.10 | |
| No of residents * House location in the village | 1.19 | 0.28 | |
| House location in the village (center/edge) | 0.32 | 0.57 | |
| Cooking inside houses (yes/no) | 0.20 | 0.65 | |

**Supplementary Table 4**: Statistical summary of the effect of housing conditions and local environmental variables on the number of malaria cases in Abulo.

| **Variable** | **F Ratio** | **Prob > F** |
| --- | --- | --- |
| House shape (square/circle) | 0.038 | 0.96 |
| Wall material (wood/mud/painted) | 0.18 | 0.84 |
| Wall condition (good/poor) | 0.17 | 0.68 |
| Door material (curtain/wood/metal) | 2.67 | 0.074 |
| Door condition (good/poor) | 0.46 | 0.71 |
| Number of windows | 0.027 | 0.87 |
| Roof material (grass/metal) | 0.013 | 0.91 |
| Roof condition (good/poor) | 0.0076 | 0.93 |
| Presence or absence of eaves | 0.32 | 0.57 |
| Livestock ownership (yes/no) | 0.023 | 0.88 |
| Cooking inside or outside of houses | 0.39 | 0.53 |
| Breeding site within 50 m radius (present/absent) | 2.65 | 0.11 |
| Household size (No of occupants) | 6.70 | 0.011 |
| House location in the village (center/edge) | 1.07 | 0.30 |
| Net use (proper use/no use) | 0.15 | 0.70 |
| Net use * House location in the village | 0.13 | 0.72 |

**Supplementary Table 5**: Statistical summary of the effect of housing conditions and local environmental variables on the number of malaria cases in Magge.

| **Variable** | **F Ratio** | **Prob > F** |
| --- | --- | --- |
| House shape (square/circle) | 0.33 | 0.72 |
| Wall material (wood/mud/painted) | 0.58 | 0.45 |
| Wall condition (good/poor) | 2.53 | 0.11 |
| Door material (curtain/wood/metal) | 0.35 | 0.79 |
| Door condition (good/poor) | 2.85 | 0.062 |
| Number of windows | 0.39 | 0.53 |
| Window coverage (none/metal/wood) | 1.01 | 0.39 |
| Roof condition (good/poor) | 2.88 | 0.092 |
| Presence or absence of eaves | 0.50 | 0.48 |
| Livestock ownership (yes/no) | 3.12 | 0.080 |
| Cooking inside or outside of houses | 1.34 | 0.25 |
| Breeding site within 50 m radius (present/absent) | 0.39 | 0.54 |
| Household size (No of occupants) | 1.40 | 0.24 |
| House location in the village (center/edge) | 5.75 | 0.018 |
| Household size * House location in the village | 1.61 | 0.21 |
| Net use (proper use/no use) | 1.22 | 0.27 |
| Net use * House location in the village | 0.048 | 0.83 |
